# Predictive value of the age, creatinine, and ejection fraction score in patients with myocardial infarction with nonobstructive coronary arteries

**DOI:** 10.1002/clc.23650

**Published:** 2021-06-01

**Authors:** Side Gao, Wenjian Ma, Sizhuang Huang, Xuze Lin, Mengyue Yu

**Affiliations:** ^1^ Department of Cardiology Fuwai Hospital, National Center for Cardiovascular Diseases, Chinese Academy of Medical Sciences and Peking Union Medical College Beijing China

**Keywords:** ACEF score, cardiovascular outcomes, myocardial infarction with nonobstructive coronary arteries (MINOCA), risk stratification

## Abstract

**Background:**

Little is known about risk stratification in patients with myocardial infarction with nonobstructive coronary arteries (MINOCA). We investigated whether the age, creatinine, and ejection fraction (ACEF) score (age [years]/ejection fraction [%] + 1 [if creatinine >176 μmol/L]) might predict long‐term outcomes after MINOCA.

**Hypothesis:**

The ACEF score enables accurate risk prediction in patients with MINOCA.

**Methods:**

A total of 1179 patients with MINOCA were enrolled and divided based on their ACEF score tertile levels. The primary endpoint was a composite of major adverse cardiovascular events (MACE), including all‐cause death, nonfatal MI, nonfatal stroke, revascularization, and hospitalization for unstable angina or heart failure. Kaplan–Meier and Cox regression analyses were performed. Discrimination was defined as the area under the curve (AUC) using receiver operating characteristic analysis.

**Results:**

During the median follow‐up of 41.7 months, patients with MINOCA with higher ACEF score tertiles had a significantly higher incidence of MACE (6.3%, 12.5%, and 23.8%, respectively; *p* < .001). The adjusted risk of MACE increased with the rising ACEF score tertiles (1st tertile as reference; 2nd tertile: HR 2.70, 95% CI: 1.38–5.29, *p* = .004; and 3rd tertile: HR 5.35, 95% CI: 2.72–10.51, *p* < .001). Moreover, an elevated ACEF score was closely associated with an increased risk of MACE overall (HR 4.23, 95% CI: 3.37–5.30, *p* < .001) and in subgroups (all *p* < .05). The ACEF score also yielded a good predictive value (AUC 0.79) for MACE.

**Conclusion:**

Elevated ACEF scores were strongly associated with a poor prognosis after MINOCA. This simple and valid risk score may facilitate risk stratification and decision making in the population with MINOCA.

## 1 INTRODUCTION

Acute myocardial infarction (AMI) remains a leading contributor to high rates of mortality and morbidity worldwide, despite advances in medical and interventional therapies^.^ ^1^ Recently, a distinct population with myocardial infarction with nonobstructive coronary arteries (MINOCA) has been increasingly recognized with the widespread use of coronary angiography^.^ ^2^, ^3^ As reported, MINOCA occurs in 5%–10% of all patients with AMI and disproportionately affects younger patients and females in comparison to those with obstructive coronary artery disease (CAD)^.^ ^4^ The underlying pathophysiological mechanisms of MINOCA are varied and may include plaque rupture or erosion, spasm, thromboembolism, dissection, and supply/demand mismatch. Other nonischemic diseases, such as myocarditis and Takotsubo syndrome, may also mimic the presentation of MINOCA.^5^ Several studies have confirmed that patients with MINOCA are still at considerable risk for long‐term adverse cardiovascular (CV) events, highlighting the opportunities to improve prognosis for this specific population.^6^, ^7^, ^8^, ^9^, ^10^, ^11^ In this context, accurate and early risk stratification is of crucial necessity and has profound implications in the management of MINOCA.

The prognosis after AMI has been addressed for decades, and many risk scores are currently in use.^12^, ^13^ Of these, the age, creatinine, and ejection fraction (ACEF) score has drawn more attention due to its accuracy and clinical applicability in patients with AMI or all‐comers undergoing percutaneous coronary intervention (PCI).^14^, ^15^, ^16^, ^17^, ^18^, ^19^ However, the outcomes of MINOCA remain largely unreported, and data regarding the risk factors for MINOCA are scarce. Here, we hypothesized that the ACEF score might be a simple and user‐friendly tool for risk stratification in patients with MINOCA. To verify this, we investigated the association between the ACEF score and long‐term outcomes after MINOCA and further explored whether the ACEF score might provide significant prognostic information in this specific population.

## 2 METHODS

### 2.1 Study population

**FIGURE 1:**
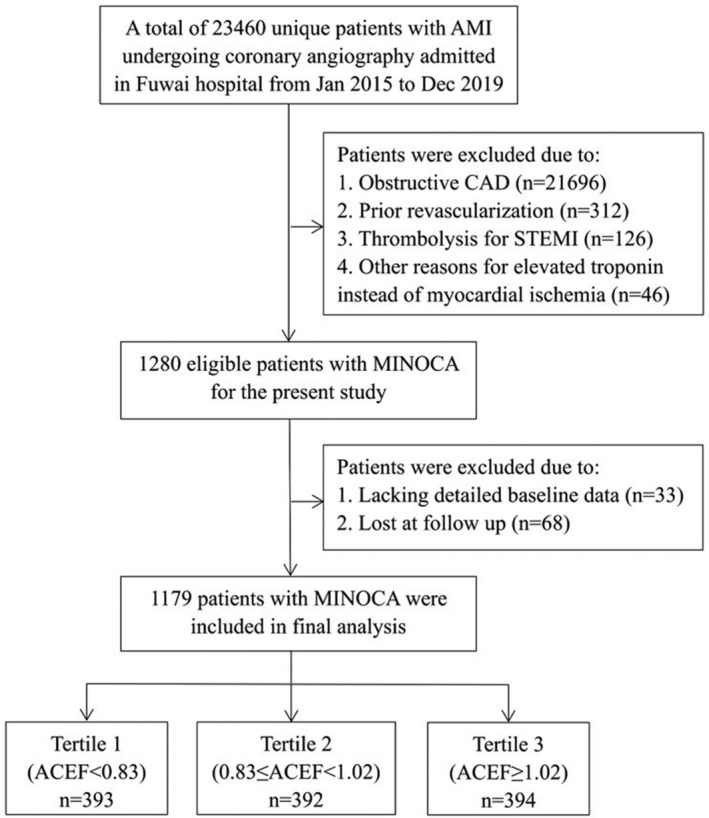
Study flowchart

The present study was a single‐center, prospective and observational cohort study of MINOCA. From January 2015 to December 2019, a total of 23 460 unique patients with AMI who underwent coronary angiography were consecutively admitted to Fuwai Hospital, including non‐ST‐segment elevation myocardial infarction (NSTEMI) and ST‐segment elevation myocardial infarction (STEMI). Patients were identified as having MINOCA if the confirmed diagnosis met the 4th universal definition of AMI^20^ and the coronary angiogram did not show a stenosis of ≥50% in the epicardial coronary arteries.^2^ The exclusion criteria were as follows: (1) obstructive CAD (*n* = 21 696); (2) previous revascularization (*n* = 312); (3) thrombolytic therapy for STEMI since the severity of coronary lesions may be changed after thrombolysis (*n* = 126); (4) alternate explanations for elevated troponin rather than coronary‐related diseases (*n* = 46; e.g., heart failure, myocarditis, pulmonary embolism, and Takotsubo syndrome); (5) lack of detailed baseline data (*n* = 33); and (6) being lost to follow‐up (*n* = 68). Finally, 1179 patients with MINOCA were enrolled in the analysis (Figure 1). All patients were prescribed evidence‐based optimal medical therapies.^12^, ^13^ This study was approved by the Ethics Committee of Fuwai Hospital and was conducted in accordance with the Declaration of Helsinki. All enrolled subjects provided written informed consent.

### 2.2 Data collection

Baseline characteristics with regard to the demographic, clinical, and laboratory data were obtained from in‐person interviews and medical records. Body mass index (BMI) was calculated as weight (kg) divided by height (m)^2^. Concentrations of fasting blood glucose (FBG), creatinine, low‐density lipoprotein cholesterol (LDL‐C) and high‐sensitivity C‐reactive protein (hs‐CRP) were measured with an automatic biochemistry analyzer. The N‐terminal pro‐B‐type natriuretic peptide (NT‐proBNP) at admission and peak cardiac troponin I (TnI) values were recorded. The left ventricular ejection fraction (LVEF) was measured using the biplane Simpson method with echocardiography. The ACEF score was calculated upon admission using the following formula: age (years)/LVEF (%) + 1 (if creatinine >176 μmol/L).^21^ In the case of multiple assessments, the basal values of LVEF and creatinine during the first 72 h after the index MINOCA were recorded. The Global Registry of Acute Coronary Event (GRACE) score was also calculated at admission as previously described.^22^

### 2.3 Definitions and outcomes

Diabetes mellitus (DM) was defined as having a history of DM or newly diagnosed DM with a FBG ≥7.0 mmol/L or 2 h plasma glucose ≥11.1 mmol/L.^23^ Hypertension was defined as a repeated blood pressure ≥140/90 mmHg or current use of antihypertensive drugs. Dyslipidemia was defined as LDL‐C ≥3.4 mmol/L, high‐density lipoprotein cholesterol <1.0 mmol/L, triglyceride ≥1.7 mmol/L or patients with a medical history of dyslipidemia.^24^

The primary study endpoint was a composite of major adverse cardiovascular events (MACE), including all‐cause death, nonfatal MI, revascularization, nonfatal stroke, and hospitalization for unstable angina (UA), or heart failure (HF). The MACE was assessed as time to first event. The secondary endpoints included each component of MACE and the composite “hard” endpoint of death, nonfatal MI, revascularization, and nonfatal stroke. Reinfarction was diagnosed according to the 4th universal definition of MI.^20^ Revascularization was performed at the operator's discretion due to recurrent ischemia and progression of coronary lesions. Stroke was defined by the presence of neurological dysfunction and vascular brain injury caused by cerebral ischemia or hemorrhage.^25^ Hospitalization for UA or HF reflected the clinical status and quality of life after AMI. Specifically, UA was diagnosed if the symptoms worsened with an increase in severity or length of anginal attacks.^12^ HF was defined as typical symptoms and evidence of a structural or functional cardiac abnormality.^25^ Patients were regularly followed up at clinics or through telephone contact by a team of independent research physicians or nurses blinded to the purpose of this study. All study endpoints were confirmed by at least two professional cardiologists.

### 2.4 Statistical analysis

Continuous variables were expressed as the mean ± standard deviation or median with interquartile range in cases of skewed distribution. Categorical variables are presented as numbers with percentages. Differences were assessed using analysis of variance or the Kruskal–Wallis H test for continuous variables and Pearson's *χ* ^2^ or Fisher's exact test for categorical variables. Survival curves showing the cumulative incidence of MACE among groups were conducted using Kaplan–Meier analysis and compared by the log‐rank test. Univariate and multivariate Cox proportional regression analyses were performed to identify the association between the ACEF score and event risk. Clinically relevant and prognosis‐related variables among groups were enrolled in the multivariate Cox model, including sex; MI type (NSTEMI or STEMI); and presence of hypertension, diabetes or dyslipidemia. The hazard ratios (HRs) with 95% confidence intervals (CIs) were calculated. The accuracy of predictors for MACE was defined by the area under the curve (AUC) using receiver operating characteristic (ROC) curve analysis. The AUC values were interpreted as follows: Negligible (≤0.55), small (0.56–0.63), moderate (0.64–0.70), and strong (≥0.71).^26^ The difference in discrimination between the ACEF score and GRACE score was appraised using DeLong's test^27^ with MedCalc V.11.4 (MedCalc Inc., Ostend, Belgium). All tests were 2‐tailed, and *p* < .05 was considered statistically significant. Unless stated otherwise, most of the analyses were performed with SPSS V.22.0 (SPSS Inc., Chicago, IL, USA).

## 3 RESULTS

### 3.1 Baseline characteristics

Patients with MINOCA were divided according to the tertile ACEF score (tertile 1: ACEF <0.83; tertile 2: 0.83≤ ACEF <1.02; and tertile 3: ACEF ≥1.02) (Figure 1). The ACEF score was normally distributed in all patients (Supplementary Figure 1). As shown in Table 1, patients with higher ACEF scores were older and more often female. They were more likely to receive emergent coronary angiography and had a higher prevalence of hypertension, diabetes, and previous MI. They also had lower BMI; higher Killip class; higher GRACE score; lower LVEF; and higher FBG, creatinine, hs‐CRP, NT‐proBNP, and peak TnI values. The medication at discharge did not differ significantly among the groups. We further compared the characteristics among patients with or without MACE (Supplementary Table 1) and found that those who developed MACE had more risk profiles at baseline. In this regard, the ACEF score approximately mirrors the general CV risk profiles and burden of comorbidities in patients with MINOCA.

**TABLE 1:** Baseline characteristics and clinical outcomes of MINOCA patients based on the ACEF score tertiles

	Low ACEF score (*n* = 393)	Medium ACEF score (*n* = 392)	High ACEF score (*n* = 394)	*p* value
Female, *n* (%)	56 (14.2%)	113 (28.8%)	143 (36.2%)	<.001
Age, years	43.8 ± 7.8	56.7 ± 5.1	66.4 ± 8.6	<.001
BMI, kg/m^2^	25.8 ± 3.5	25.5 ± 3.5	25.0 ± 4.2	.013
STEMI, *n* (%)	165 (41.9%)	154 (39.2%)	156 (39.5%)	.178
Emergent angiography, *n* (%)	30 (7.6%)	58 (14.7%)	71 (18.0%)	<.001
Past history				
Hypertension	172 (43.7%)	219 (55.8%)	239 (60.6%)	<.001
Diabetes	38 (9.6%)	63 (16.0%)	86 (21.8%)	<.001
Dyslipidemia	221 (56.2%)	231 (58.9%)	232 (58.8%)	.125
Previous MI	10 (2.5%)	25 (6.3%)	23 (5.8%)	.027
Killip class≥2, *n* (%)	11 (2.7%)	23 (5.8%)	55 (13.9%)	<.001
LVEF (%)	63.4 ± 5.5	61.6 ± 4.7	56.4 ± 9.3	<.001
Clinical risk scores				
ACEF score	0.69 ± 0.11	0.92 ± 0.05	1.39 ± 2.26	<.001
GRACE score	114.9 ± 23.6	139.1 ± 25.5	167.6 ± 27.2	<.001
Laboratory tests				
FBG, mmol/L	5.41 ± 1.39	5.77 ± 1.71	5.93 ± 1.92	<.001
Creatinine, μmol/L	76.0 ± 12.9	79.4 ± 13.1	84.9 ± 24.1	<.001
LDL‐C, mmol/L	2.31 ± 0.77	2.28 ± 0.73	2.28 ± 0.77	.856
hs‐CRP, mg/L	3.72 ± 3.86	3.80 ± 3.76	4.37 ± 4.35	.044
NT‐proBNP, pg/ml	204 (72, 515)	375 (113, 673)	649 (216, 2094)	<.001
Peak TnI, ng/ml	1.5 (0.2, 3.1)	3.1 (0.7, 6.5)	4.4 (0.9, 15.1)	<.001
Medication at discharge				
DAPT	364 (92.6%)	367 (93.6%)	358 (90.8%)	.316
Statin	372 (94.6%)	375 (95.6%)	384 (97.4%)	.250
ACEI or ARB	246 (62.5%)	253 (64.5%)	260 (65.9%)	.608
Beta‐blocker	294 (74.8%)	281 (71.6%)	285 (72.3%)	.880
Clinical outcomes				
MACE	25 (6.3%)	49 (12.5%)	94 (23.8%)	<.001
Death, nonfatal MI, nonfatal stroke or revascularization	13 (3.3%)	26 (6.6%)	58 (14.7%)	<.001
All‐cause death	2 (0.5%)	5 (1.2%)	11 (2.7%)	<.001
Nonfatal MI	7 (1.7%)	9 (2.2%)	25 (6.3%)	.001
Revascularization	8 (2.0%)	18 (4.5%)	20 (5.0%)	.045
Nonfatal stroke	1 (0.2%)	3 (0.7%)	8 (2.0%)	.018
Hospitalization for UA	13 (3.3%)	31 (7.9%)	27 (6.8%)	.012
Hospitalization for HF	5 (1.2%)	5 (1.2%)	38 (9.6%)	<.001

*Note*: Patients were divided according to the tertile levels of the Age, Creatinine, and Ejection Fraction (ACEF) score (Tertile 1: ACEF <0.83, Tertile 2: 0.83 ≤ ACEF <1.02, Tertile 3: ACEF ≥1.02).

Abbreviations: ACEI, angiotensin‐converting enzyme inhibitor; ARB, angiotensin receptor antagonist; BMI, body mass index; DAPT, dual anti‐platelet therapy; FBG, fasting blood glucose; GRACE, Global Registry of Acute Coronary Event; HF, heart failure; hs‐CRP, high‐sensitive C‐reactive protein; LDL‐C, low density lipoprotein‐cholesterol; LVEF, left ventricular ejection fraction; MACE, major adverse cardiovascular events; MI, myocardial infarction; NT‐proBNP, N‐terminal pro‐B‐type natriuretic peptide; STEMI, ST‐segment elevation myocardial infarction; TnI, Troponin I; UA, unstable angina.

### 3.2 Relationship between the ACEF score and CV outcomes

**FIGURE 2:**
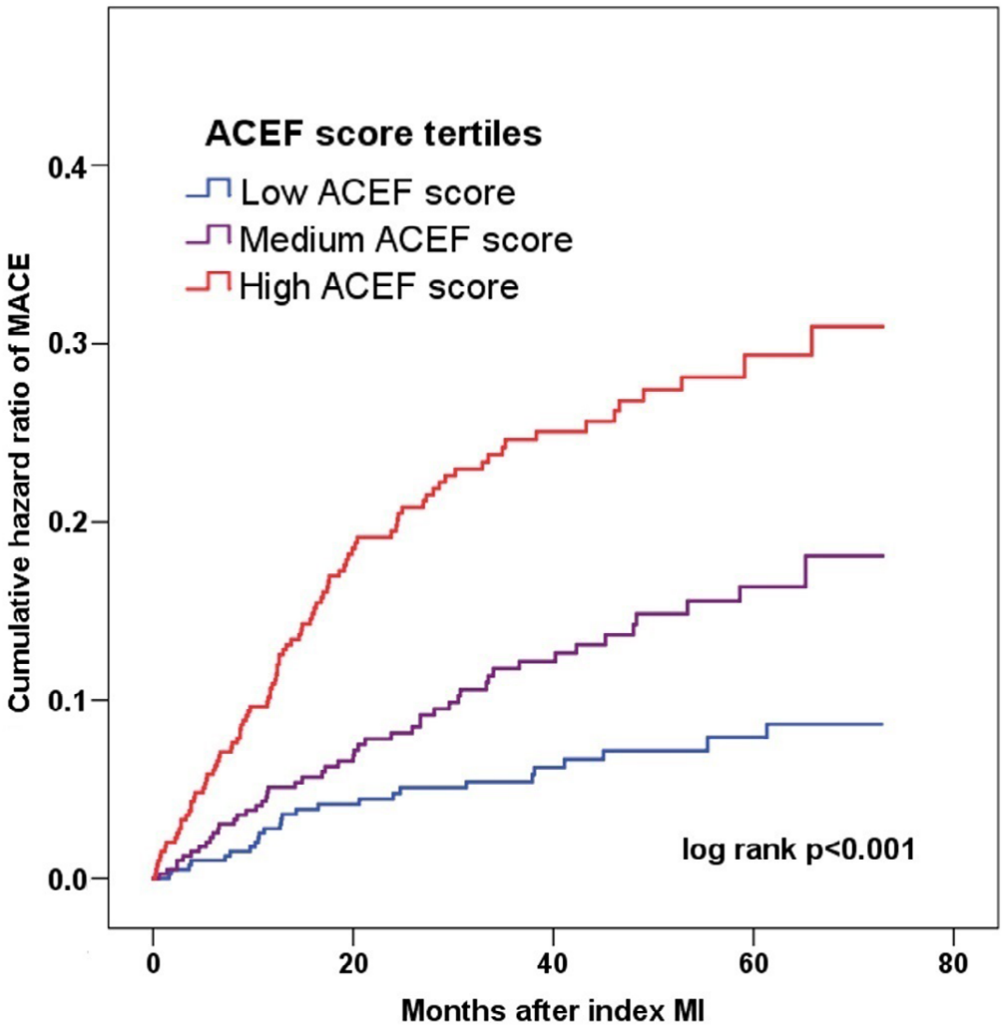
Cumulative incidence of MACE in patients with MINOCA stratified by the age, creatinine, and ejection fraction (ACEF) score tertiles. Low ACEF score: ACEF <0.83, Medium ACEF score: 0.83 ≤ ACEF <1.02, High ACEF score: ACEF ≥1.02

Patients with higher ACEF tertiles had a higher incidence of MACE (6.3%, 12.5%, and 23.8%, respectively; *p* < .001) and other secondary endpoints (all *p* < .05) after MINOCA (Table 1). After multivariate adjustment, the risk of MACE still increased in parallel with the ACEF score tertiles (1st tertile as reference; 2nd tertile: HR = 2.70, 95% CI: 1.38–5.29, *p* = .004; and 3rd tertile: HR = 5.35, 95% CI: 2.72–10.51, *p* < .001). The risk of the composite endpoint of death, MI, revascularization or stroke also increased with the rising ACEF tertiles (Table 2). The Kaplan–Meier curves indicated that patients with higher ACEF tertile levels had a higher cumulative incidence of MACE (log rank *p* < .001) (Figure 2). Moreover, an elevated ACEF score was significantly associated with an increased risk of MACE overall (for a 1 SD increase in the ACEF score, adjusted HR = 4.23, 95% CI: 3.37–5.30, *p* < .001) (Table 2). In the subgroup analysis, the ACEF score remained a robust predictor of prognosis in subsets of patients stratified by sex, age, BMI, MI type, hypertension, diabetes, dyslipidemia, estimated glomerular filtration rate (eGFR) and LVEF level (all *p* < .05) (Supplementary Figure 2).

**TABLE 2:** Association between the ACEF score level and event risk

Groups	Univariate Cox analysis		Multivariate Cox analysis	
	HR (95% CI)	*p* value	HR (95% CI)	*p* value
MACE				
ACEF, per 1SD increase	4.11 (3.33–5.08)	<.001	4.23 (3.37–5.30)	<.001
Low ACEF score	1 (reference)	…	1 (reference)	…
Medium ACEF score	2.10 (1.30–3.41)	.002	2.70 (1.38–5.29)	.004
High ACEF score	4.40 (2.83–6.85)	<.001	5.35 (2.72–10.51)	<.001
Death, nonfatal MI, nonfatal stroke or revascularization				
ACEF, per 1SD increase	4.33 (3.36–5.58)	<.001	4.45 (3.42–6.03)	<.001
Low ACEF score	1 (reference)	…	1 (reference)	…
Medium ACEF score	2.11 (1.08–4.12)	.027	3.06 (1.22–7.68)	.017
High ACEF score	5.46 (3.00–9.92)	<.001	4.51 (1.92–10.61)	.001

*Note*: HR was adjusted for gender, MI type (NSTEMI or STEMI), hypertension, diabetes and dyslipidemia in the multivariate Cox model.

Abbreviations: CI, confidence interval; HR, hazard ratio; MACE, major adverse cardiovascular events; MI, myocardial infarction; SD, standard deviation.

### 3.3 Predictive values of the ACEF score for MACE

**FIGURE 3:**
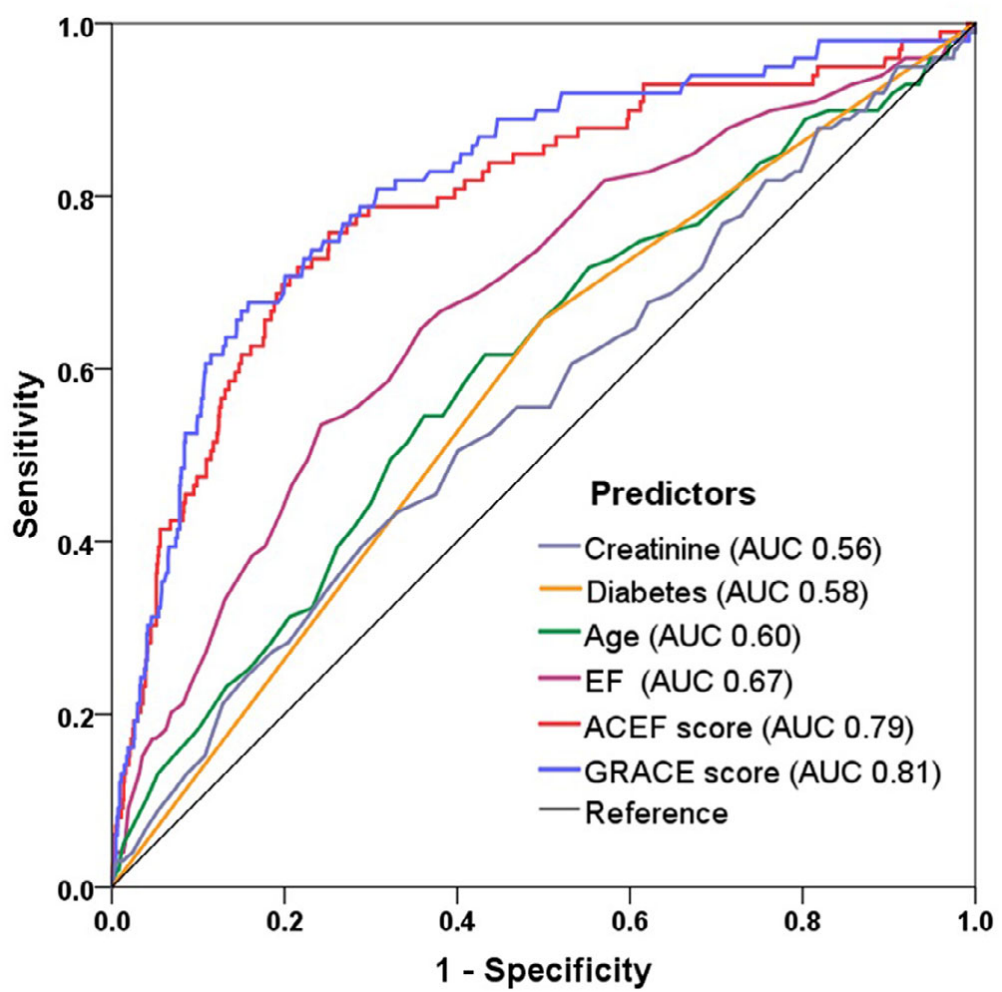
Predictive value of the risk factors and risk scores for MACE. Receiver operating characteristic curves showing the predictive value of age, creatinine, ejection fraction (EF), diabetes, ACEF score, and GRACE score for MACE in patients with MINOCA. AUC, area under the curve; GRACE, Global Registry of Acute Coronary Event; MACE, major adverse cardiovascular events; MINOCA, myocardial infarction with nonobstructive coronary arteries

Univariate Cox regression analysis showed several clinically relevant risk factors. Of these, only older age, diabetes, LVEF, and creatinine were identified as independent predictors of MACE after multivariate adjustment (Supplementary Table 2). In the ROC curve analysis (Figure 3), the predictive values of these four variables were moderate, whereas the ACEF score (AUC 0.79) and GRACE score (AUC 0.81) yielded better accuracy for the prediction of MACE. There was no significant difference in discrimination between the ACEF and GRACE scores (*p* > .05 by Delong's test).

## 4 DISCUSSION

Our data, for the first time, verified the prognostic value of the ACEF score in patients with MINOCA and proved that elevated ACEF scores were strongly associated with poor outcomes after MINOCA. It might be reasonable to use the ACEF score as an efficient tool for risk stratification in the contemporary real‐world management of MINOCA.

With the widespread use of coronary angiography in the management of AMI, there has been an increasing awareness that a proportion of patients with AMI have no evidence of obstructive CAD. These patients with MINOCA have a variety of underlying causes for elevated troponin, including coronary (e.g., plaque rupture and spasm) and noncoronary causes (e.g., myocarditis). Recently, the term MINOCA has been primarily used to describe patients with coronary‐related ischemia.^2^, ^3^, ^4^, ^5^ We adopted these criteria and excluded those with noncoronary artery diseases. Nearly 5.1% of patients with AMI were identified as having MINOCA in our study, which is close to the estimated prevalence of 6% in a systematic review.^4^ As reported, approximately one‐third of patients with MINOCA presented with a STEMI. They were more likely to be younger, female, and have fewer comorbidities than individuals with obstructive AMI.^4^ We described the clinical profiles of patients with MINOCA as well, which were generally in line with previous studies. Nevertheless, the percentage of women was relatively low in our cohort, possibly due to the large proportion of male patients with AMI treated in our center and a lower rate for women who receive angiography. Given the potential selection bias in single‐center studies, future nationwide registry cohorts of patients with MINOCA are needed to validate our findings. It seems that patients with MINOCA have better outcomes than those with AMI and significant CAD; however, their long‐term prognosis is not trivial, especially considering that they are younger and have fewer CV risk factors.^2^, ^3^, ^4^, ^5^ Recent studies have revealed that these patients are still at considerable risk for 1‐year mortality and the occurrence of MACE after MINOCA.^6^, ^7^, ^8^, ^9^, ^10^, ^11^ Consistently, we found that 1.5% of patients with MINOCA died and 14.2% of them experienced MACE during the median follow‐up of 3.5 years, highlighting the potential opportunities to improve healthcare for this population.

Over the decades, a number of risk scores have been developed to predict adverse events after AMI, and they are still currently being used in daily practice. Of these, the ACEF score, using only three variables (age, LVEF, and creatinine), was first proposed and validated by Ranucci et al. in 2009 to assess mortality risk in elective cardiac operations^21^ and was later updated to the ACEF II score in 2018.^28^ Although it was originally designed for patients undergoing cardiac surgeries, recent studies have proven the prognostic power of the ACEF score in patients with AMI, suggesting that an elevated ACEF score was significantly associated with a poor outcome after AMI.^14^, ^15^ The ACEF score was also correlated with the risk of MACE in all‐comers treated with PCI^16^, ^17^, ^18^, ^19^ and particularly in patients with challenging lesions, such as bifurcation lesions,^17^ heavy calcification,^18^ and chronic total occlusion.^19^ Additionally, the clinical Synergy between PCI with TAXUS and Cardiac Surgery (SYNTAX) score also incorporates the ACEF score by multiplying the original SYNTAX score to take clinical characteristics into account.^29^ Given its good performance, the European Society of Cardiology (ESC) guidelines on myocardial revascularization recommended the ACEF score for risk stratification in 2010^30^ and further confirmed its utility in their 2014^31^ and 2018 updates.^32^

The present study extended the utility of the ACEF score to patients with MINOCA who received evidence‐based optimal medical therapies in the largest CV center in China. We found that the ACEF score independently predicted long‐term outcomes in patients with MINOCA. Interestingly, the three variables in the ACEF score (age, LVEF, and creatinine) were also the most powerful predictors of MACE in our cohort. We believe that the ACEF score might be particularly suitable for the population with MINOCA, since they have fewer comorbidities and no coronary obstructions, thereby avoiding the necessity of assessing lesion characteristics and incorporating too many variables in a risk model. More importantly, the ACEF score yielded a discrimination similar to that of the GRACE score. Actually, in comparison to more complex risk models, the ACEF score is extremely simple, practical, easy to calculate, and has good clinical applicability. The ACEF score also has the advantage of incorporating readily available variables.^21^ These factors are objectively measurable and thus may eliminate the bias introduced by personal interpretation of categorical variables and coronary angiograms, which may be prone to interobserver variability.^33^ Furthermore, the ACEF score follows the “law of parsimony,” that is, risk factors should not be multiplied beyond necessity.^34^ This may avoid the problem of overfitting and multicollinearity induced by incorporation of too many independent variables in a risk model, especially in populations with low numbers of events.^21^, ^35^ Other studies have also found that the accuracy of the ACEF score was similar to that of conventional risk scores or models.^14^, ^15^ However, it was not our intention to conclude that a novel simple risk model is superior to existing risk scores. Instead, these well‐established scores have been verified and used for years, and they all have a reputation for statistical soundness and clinical usefulness. Even though the ACEF score performed well in predicting outcomes after MINOCA, this risk stratification tool still needs long‐term validation in different cohorts.

In clinical practice, the prognosis of patients with MINOCA should not be ignored, because they are still at risk of developing future adverse events. This highlights the necessity of early and valid risk stratification for this population. The ACEF score allows for accurate prediction of prognosis in patients with MINCOA and may further assist in clinical decision making. Physicians may use the ACEF score as a simple and user‐friendly tool and give special attention to those at high risk and thus tailor targeted treatment.

Several limitations should be mentioned. First, the present data were derived from a single center, and the sample size was limited. Although few studies have enrolled more than a 1000 subjects, as we did, our findings still warrant verification by future nationwide cohort studies of patients with MINOCA. Second, we did not capture and record the exact mechanism for every patient with MINOCA. The association between the etiology of MINOCA and outcomes should be further explored. Third, although multivariate adjustment and subgroup analyses were performed, there might be other residual confounding factors that may have affected the prognosis. Fourth, the measurements of LVEF and creatinine after discharge would help to assess changes in cardiac and renal function during follow‐up, but these data were not available in the present study. Furthermore, the proposed ACEF score risk categories need to be tested in an external validation cohort.

## 5 CONCLUSION

The ACEF score provided significant prognostic information in patients with MINOCA. Assessment of the ACEF score may help to identify patients at high risk of developing MACE after MINOCA and may further facilitate pre‐emptive clinical decision making.

## CONFLICT OF INTEREST

The authors declare that they have no competing interests.

## AUTHOR CONTRIBUTIONS

Side Gao conceived and designed the study. Side Gao, Wenjian Ma, Sizhuang Huang, and Xuze Lin performed data analysis and interpretation. Side Gao drafted the manuscript. Mengyue Yu reviewed and gave final approval of the version to be published. All authors read and approved the final manuscript.

## Supporting information


**Appendix S1**. Supporting Information.

## Data Availability

The data that support the findings of this study are available from the corresponding author upon reasonable request.
