# Supplementary material for: Predictive value of the age, creatinine, and ejection fraction score in patients with myocardial infarction with nonobstructive coronary arteries
Source: Clin Cardiol. 2021 Jun 1;44(7):1011–8. doi: 10.1002/clc.23650 (PMC8259146; doi:10.1002/clc.23650)
Supplement: Supplementary file 1 — Appendix S1. Supporting Information. [file CLC-44-1011-s001.docx]

**Supplementary Materials of the Manuscript**

**Supplementary Table 1. Clinical risk factors in patients with or without MACE.**

|  | All MINOCA  (n=1179) | With MACE  (n=168) | Without MACE  (n=1011) | P value |
| --- | --- | --- | --- | --- |
| Female, n(%) | 312 (26.5%) | 48 (28.5%) | 264 (26.1%) | 0.504 |
| Age, yrs | 57.5±11.8 | 59.7±13.2 | 55.3±11.5 | <0.001 |
| BMI, kg/m^2^ | 25.4±3.7 | 25.4±3.8 | 25.4±3.7 | 0.924 |
| STEMI, n(%) | 475 (40.2%) | 88 (52.3%) | 387 (38.2%) | 0.001 |
| Past history |  |  |  |  |
| Hypertension | 630 (53.4%) | 93 (55.3%) | 537 (53.1%) | 0.590 |
| Diabetes | 187 (15.9%) | 41 (24.4%) | 146 (14.4%) | 0.001 |
| Dyslipidemia | 686 (58.2%) | 105 (62.5%) | 581 (57.4%) | 0.221 |
| Previous MI | 58 (4.9%) | 9 (5.3%) | 49 (4.84%) | 0.114 |
| Smoking | 483 (40.9%) | 72 (42.8%) | 411 (40.6%) | 0.357 |
| Killip class≥2, n(%) | 89 (7.5%) | 21 (12.5%) | 68 (6.7%) | 0.001 |
| LVEF (%) | 60.5±7.5 | 53.0±11.7 | 61.7±5.6 | <0.001 |
| Clinical risk scores |  |  |  |  |
| ACEF score | 0.96±0.92 | 1.24±0.59 | 0.92±0.26 | <0.001 |
| GRACE score | 138.3±24.6 | 168.7±26.9 | 121.5±25.7 | <0.001 |
| Laboratory tests |  |  |  |  |
| HbA_1c_, % | 5.98±0.98 | 6.26±1.17 | 5.94±0.94 | <0.001 |
| Creatinine, μmol/L | 83.13±15.89 | 84.3±22.4 | 79.4±16.9 | 0.001 |
| LDL-C, mmol/L | 2.29±0.76 | 2.32±0.78 | 2.28±0.75 | 0.498 |
| hs-CRP, mg/L | 2.20 (1.03, 5.75) | 2.46 (1.05, 6.38) | 2.14 (1.02, 5.66) | 0.212 |
| NT-proBNP, pg/mL | 372 (112, 683) | 578 (214, 858) | 369 (107, 664) | <0.001 |
| Peak TnI, ng/mL | 3.24 (0.72, 6.51) | 4.32 (0.94, 8.13) | 3.13 (0.64, 6.27) | <0.001 |

Patients were divided based on the occurrence of major adverse cardiovascular events (MACE). BMI: body mass index, STEMI: ST-segment elevation myocardial infarction, LVEF: left ventricular ejection fraction, ACEF: Age, Creatinine, and Ejection Fraction, GRACE: Global Registry of Acute Coronary Event, HbA_1c_: glycated hemoglobin, LDL-C: low density lipoprotein-cholesterol, hs-CRP: high-sensitive C-reactive protein, NT-proBNP: N-terminal pro-B-type natriuretic peptide, TnI: Troponin I.

**Supplementary Table 2. Potential clinical risk factors for MACE in MINOCA** **patients.**

| Variables | Univariate Cox analysis | | Multivariate Cox analysis | |
| --- | --- | --- | --- | --- |
|  | HR (95% CI) | P value | HR (95% CI) | P value |
| Age | 1.06 (1.02-1.11) | 0.004 | 1.02 (1.01-1.04) | 0.012 |
| Female | 1.16 (0.83-1.62) | 0.372 | NA | … |
| BMI | 0.99 (0.95-1.03) | 0.893 | NA | … |
| STEMI | 1.42 (1.05-1.92) | 0.022 | 1.22 (0.87-1.71) | 0.237 |
| Hypertension | 1.09 (0.80-1.47) | 0.575 | NA | … |
| Diabetes | 1.86 (1.31-2.65) | 0.001 | 1.50 (1.04-2.16) | 0.030 |
| Dyslipidemia | 1.18 (0.86-1.61) | 0.300 | NA | … |
| Previous MI | 1.01 (0.51-1.99) | 0.981 | NA | … |
| LVEF | 0.92 (0.91-0.93) | <0.001 | 0.96 (0.93-0.99) | 0.012 |
| ln (NT-proBNP) | 1.39 (1.22-1.58) | <0.001 | 1.17 (0.66-2.06) | 0.587 |
| Peak TnI | 1.02 (1.01-1.03) | 0.015 | 1.01 (0.99-1.02) | 0.223 |
| Creatinine | 1.04 (1.02-1.06) | <0.001 | 1.03 (1.02-1.04) | 0.035 |

Statistically significant variables with univariate Cox analysis were further enrolled in the multivariate model. Hazard ratio (HR) for per 1 standard deviation increased in each continuous variable. NT-proBNP was natural logarithmically transformed to ln (NT-proBNP). NA: not assessed, CI: confidence interval, BMI: body mass index, STEMI: ST-segment elevation myocardial infarction, LVEF: left ventricular ejection fraction, NT-proBNP: N-terminal pro-B-type natriuretic peptide, TnI: Troponin I.


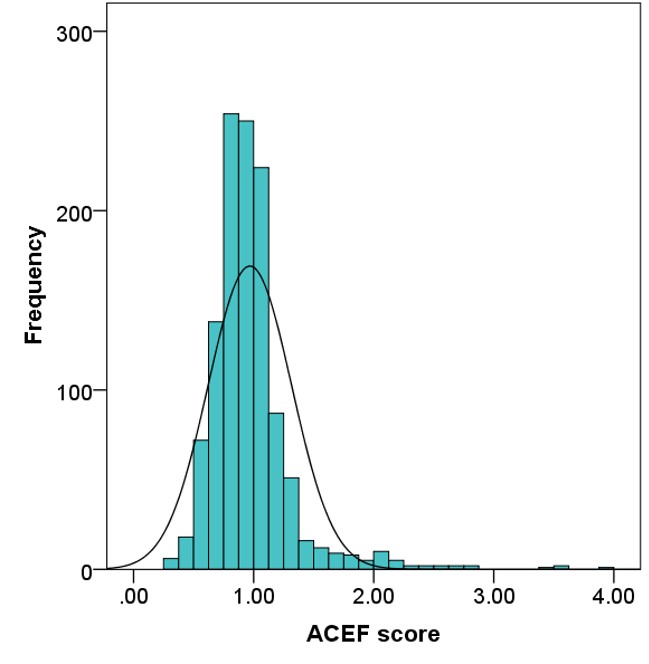


**Supplementary Figure 1. Distribution of the ACEF score.**


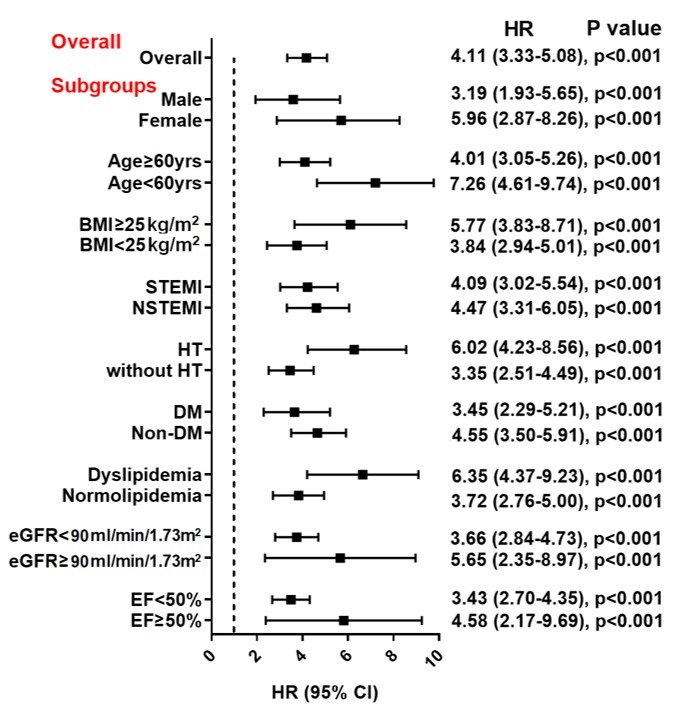


**Supplementary Figure 2. Association between the ACEF score and risk of MACE in overall and subgroups.**

Subgroup analysis for association between the ACEF score and MACE risk in patients stratified by sex, MI type, hypertension, diabetes, dyslipidemia, eGFR and LVEF level. Hazard ratio (HR) was calculated by the univariate Cox regression analysis. HR for per 1 standard deviation increased in the ACEF score. Vertical dotted line indicated the HR value of 1. BMI: body mass index, STEMI: ST-segment elevation myocardial infarction, NSTEMI: non-ST-segment elevation myocardial infarction, HT: hypertension, DM: diabetes, eGFR: estimated glomerular filtration rate, EF: ejection fraction.
